# Publisher Correction: Limits to visual representational correspondence between convolutional neural networks and the human brain

**DOI:** 10.1038/s41467-021-23110-2

**Published:** 2021-05-06

**Authors:** Yaoda Xu, Maryam Vaziri-Pashkam

**Affiliations:** 1 Psychology Department, Yale University, New Haven, CT USA; 2 Laboratory of Brain and Cognition, National Institute of Mental Health, Bethesda, MD USA

**Keywords:** Neural decoding, Object vision

Correction to: *Nature Communications* 10.1038/s41467-021-22244-7, published online 06 April 2021.

The original version of the Peer Review File associated with this Article was updated shortly after publication to redact reviewer names. This has been corrected in the PDF.

## Supplementary information

Peer Review File

