# Supplementary material for: Limits to visual representational correspondence between convolutional neural networks and the human brain
Source: Nat Commun. 2021 Apr 6;12:2065. doi: 10.1038/s41467-021-22244-7 (PMC8024324; doi:10.1038/s41467-021-22244-7)
Supplement: Supplementary file 1 — Supplemenatary Information [file 41467_2021_22244_MOESM1_ESM.pdf]

Supplementary Table 1. The CNNs and the layers examined in this study.

CNN name	Depth/Blocks	Layers	N of Layers Sampled	Sampled Layer Names and Locations (indicated in the parenthesis)
Alexnet	8	5	6	'pool1' (5), 'pool2' (9), 'pool5' (16), 'fc6' (17), 'fc7' (20), 'fc8' (23)
Cornet-S	4	42	6	'V1_outpt' (8), 'V2_output' (18), 'V4_output' (28), 'IT_output' (38), 'decoder_avgpool' (39), 'decoder_output' (42)
Densenet-201	201	709	6	'pool1' (6), 'pool2_pool' (52), 'pool3_pool' (140), 'pool4_pool' (480), 'avg_pool' (706), 'fc1000' (707)
Googlenet	22	144	6	'pool1-3x3_s2' (4), 'pool2-3x3_s2' (11), 'pool3-3x3_s2' (40), 'pool4-3x3_s2' (111), 'pool5-7x7_s1' (140), 'loss3-classifier' (142)
Inception_v3	48	316	11	'average_pooling2d_1' (29), 'average_pooling2d_2' (52), 'average_pooling2d_3' (75), 'average_pooling2d_4' (121), 'average_pooling2d_5' (153), 'average_pooling2d_6' (185), 'average_pooling2d_7' (217), 'average_pooling2d_8' (264), 'average_pooling2d_9' (295), 'avg_pool' (313), 'predictions' (314)
Inception-resnet_v2	164	825	7	'max_pooling2d_1' (12), 'max_pooling2d_2' (19), 'average_pooling2d_1' (29), 'max_pooling2d_3' (285), 'max_pooling2d_4' (648), 'avg_pool' (822), 'predictions' (823)
Mobilenet_v2	54	155	10	'block_2_project_BN' (26), 'block_4_project_BN' (43), 'block_6_project_BN' (61), 'block_8_project_BN' (78), 'block_10_project_BN' (96), 'block_12_project_BN' (113), 'block_14_project_BN' (130), 'block_16_project_BN' (148), 'global_average_pooling2d_1' (152), 'Logits' (153)
Resnet-18	18	72	6	'pool1' (6), 'res2b_relu' (20), 'res3b_relu' (36), 'res4b_relu' (52), 'pool5' (69), 'fc1000' (70)
Resnet-50	50	177	6	'max_pooling2d_1' (5), 'activation_10_relu' (37), 'activation_22_relu' (79), 'activation_40_relu' (141), 'avg_pool' (174), 'fc1000' (175)
Resnet-101	101	347	6	'pool1' (5), 'res2c_relu' (37), 'res3b3_relu' (79), 'res4b22_relu' (311), 'pool5' (344), 'fc1000' (345)
Squeezenet	18	68	5	'pool1' (4), 'pool3' (19), 'pool5' (34), 'conv10' (64), 'pool10' (66)
Vgg-16	16	41	8	'pool1' (6), 'pool2' (11), 'pool3' (18), 'pool4' (25), 'pool5' (32), 'fc6' (33),'fc7' (36), 'fc8' (39)
Vgg-19	19	47	8	'pool1' (6), 'pool2' (11), 'pool3' (20), 'pool4' (29), 'pool5' (38), 'fc6' (39),'fc7' (42), 'fc8' (45)
Xception	71	171	8	'block2_pool' (18), 'block4_pool' (42), 'block6_sepconv3_bn' (68), 'block8_sepconv3_bn' (94), 'block10_sepconv3_bn' (120), 'block12_sepconv3_bn' (146), 'avg_pool' (168), 'predictions' (169)

Supplementary Figure 1

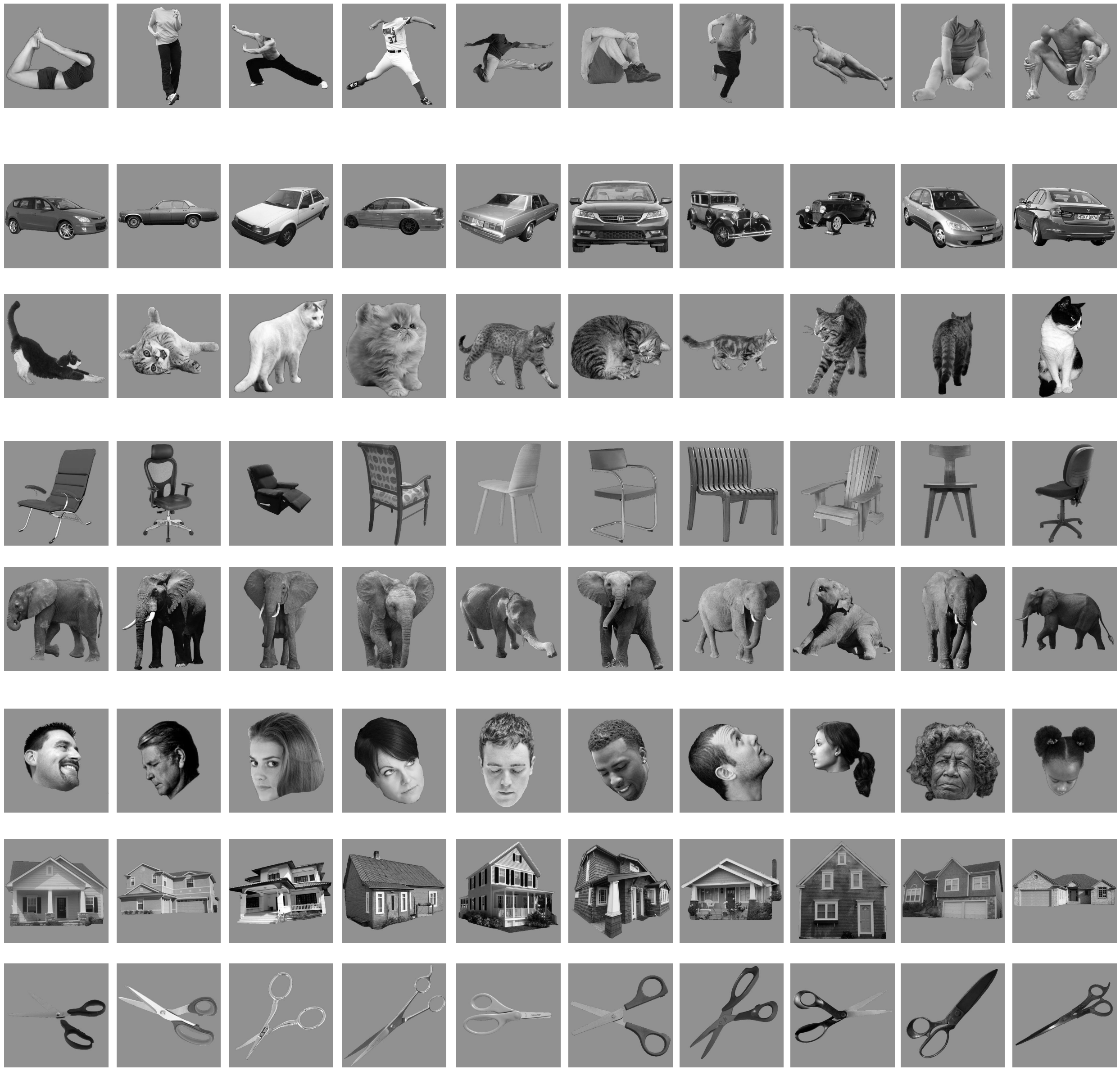

<u>Supplementary Figure 1.</u> The eight real-world object categories used in Experiments 1 to 3 and the ten exemplars appeared in each category. The exemplars within each category varied in identity, viewpoint/orientation, and pose (for the animal categories) to minimize the low-level image similarities among them.

Supplementary Figure 2

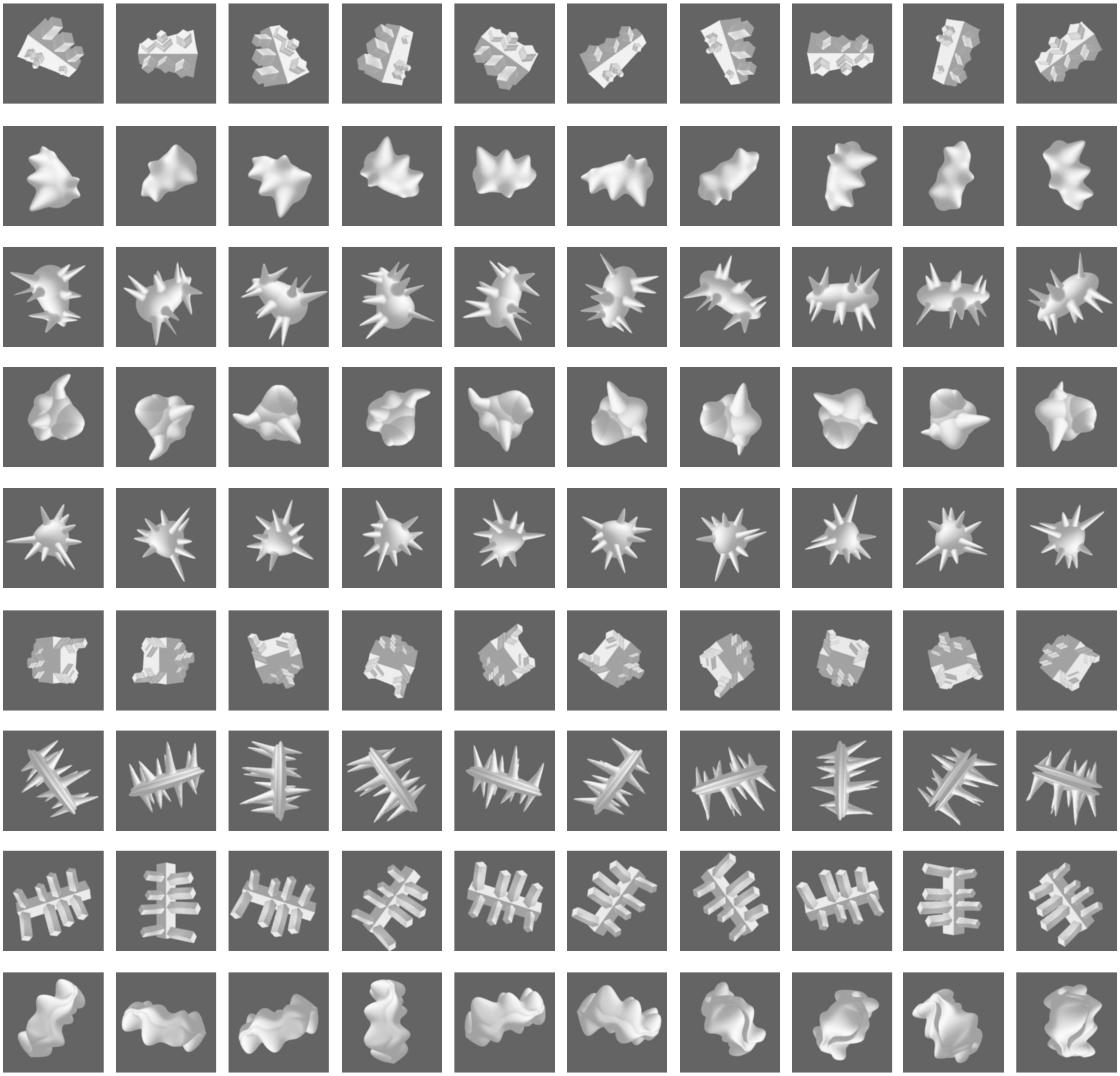

Supplementary Figure 2. The nine artificial object categories used in Experiment 3 and the ten different exemplars appeared in each category. These objects were computer-generated 3D shapes adopted from Op de Beeck et al. (2008). To match the exemplar variability present in the real-world object categories (see Supplementary Figure 1), the exemplars within each artificial object category were varied in both identity and orientation.

Supplementary Figure 3

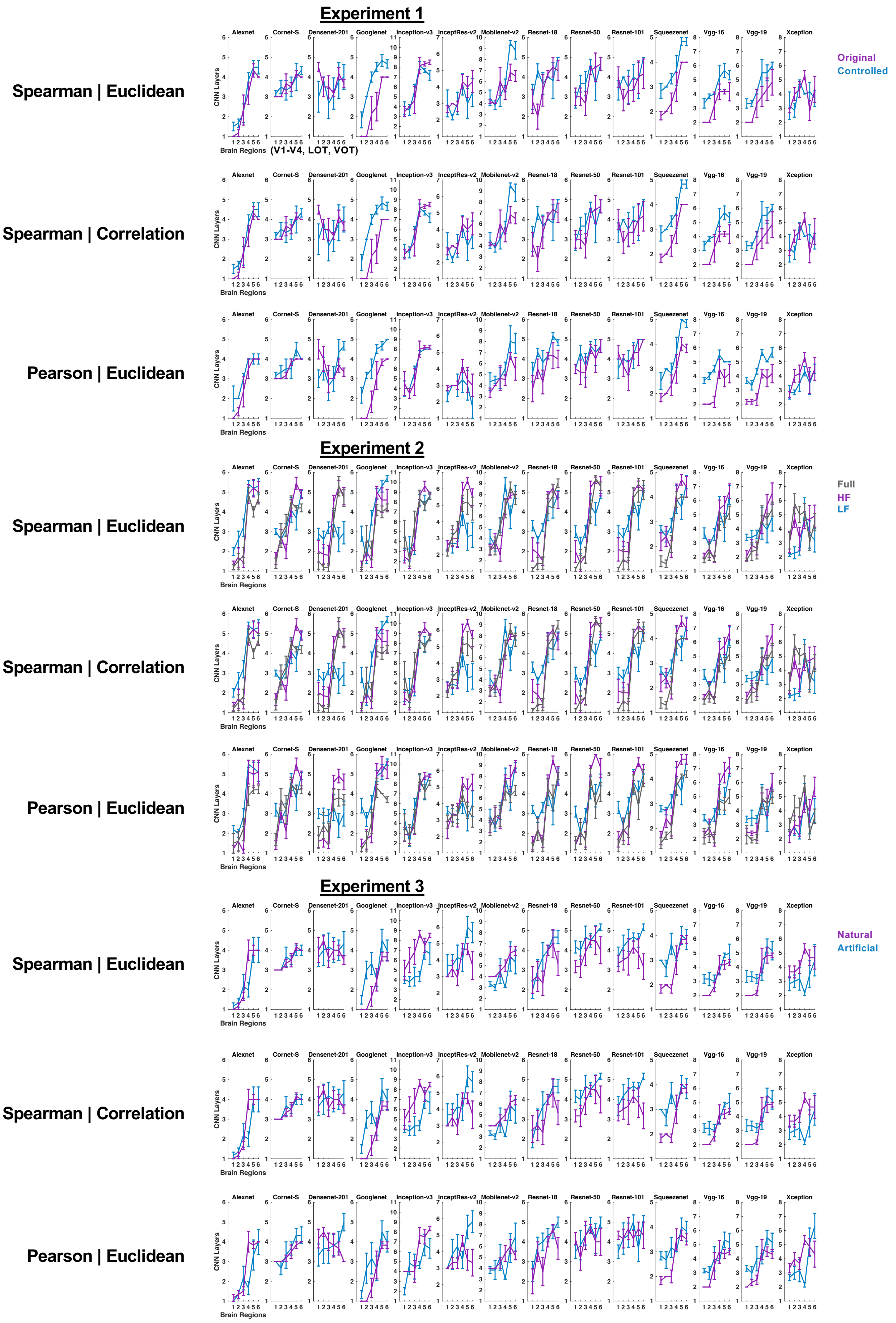

Supplementary Figure 3. Evaluating the presence of brain-CNN correspondence in their representational structures for Experiments 1 to 3. Three different measures were used, including using Spearman brain-CNN correlation with Euclidean distance measures for RDM construction (same as those shown in Figure 2), using Spearman brain-CNN correlation with correlation measures for RDM construction, and using Pearson brain-CNN correlation with Euclidean distance measures for RDM construction. *N* = 6, 10 and 6 human participants, respectively, for Experiments 1 to 3. Virtually the same results were obtained in all three measures. Error bars indicate standard errors of the means. Source data are provided as a Source Data file.

Supplementary Figure 4

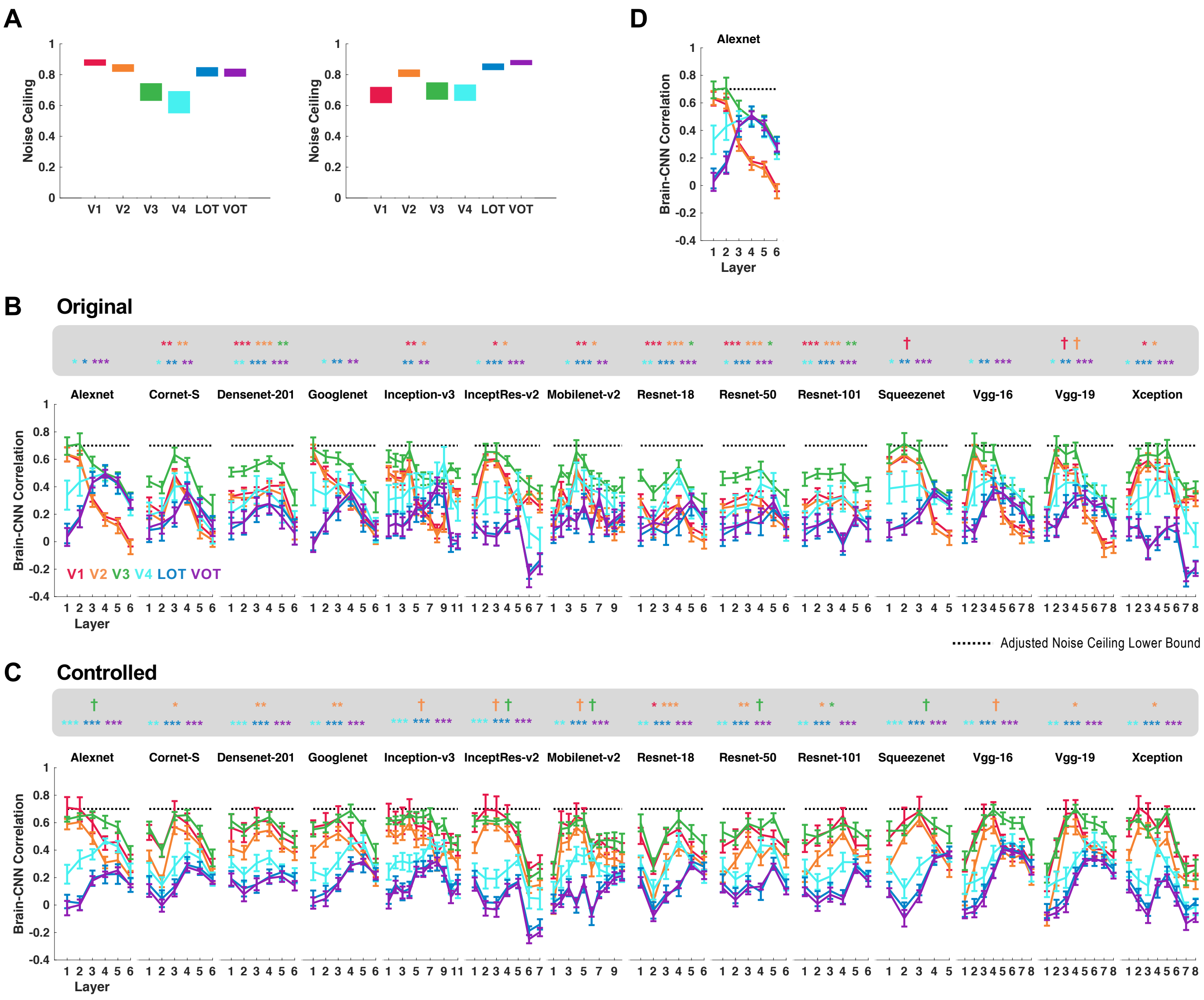

Supplementary Figure 4. Quantifying the brain-CNN correspondence in Experiment 1 with original and controlled images from real-world object categories. **(A)** The upper and lower bounds of noise ceiling of the fMRI responses for each image condition. **(B)** and **(C)** RDM correlations of each brain region with each sampled layer of each CNN for the Original and Controlled images, respectively. **(D)** RDM correlations of each brain region with each sampled layer of Alexnet for the Original images when we simulated the exact fMRI design in Alexnet by generating a matching number of randomized presentation sequences with image repetitions and then averaging CNN responses for these sequences (i.e., across 6 hypothetical participants each with 16 runs). Virtually identical Alexnet results were obtained here as those in **(B)**. $N = 6$ human participants. Because the lower bound of the noise ceiling varied somewhat among the different brain regions, for illustration purposes, while maintaining the absolute differences between the CNN and brain correlations with respect to their lower bound noise ceilings, the lower bounds of the noise ceiling from all brain regions were shifted to 0.7, and the difference between the actual noise ceiling and 0.7 was subtracted from each brain-CNN correlation value. The asterisks at the top of each plot mark the significance levels of the differences between the highest correlations (Fisher-transformed) of brain regions and CNN layers and the corresponding lower bound of the noise ceiling; one-tailed *t*-tests were used and all *p* values reported were corrected for multiple comparisons for the 6 brain regions included using the Benjamini–Hochberg procedure. Error bars indicate standard errors of the means. † $p < .1$, * $p < .05$, ** $p < .01$, *** $p < .001$. Source data are provided as a Source Data file.

Supplementary Figure 5

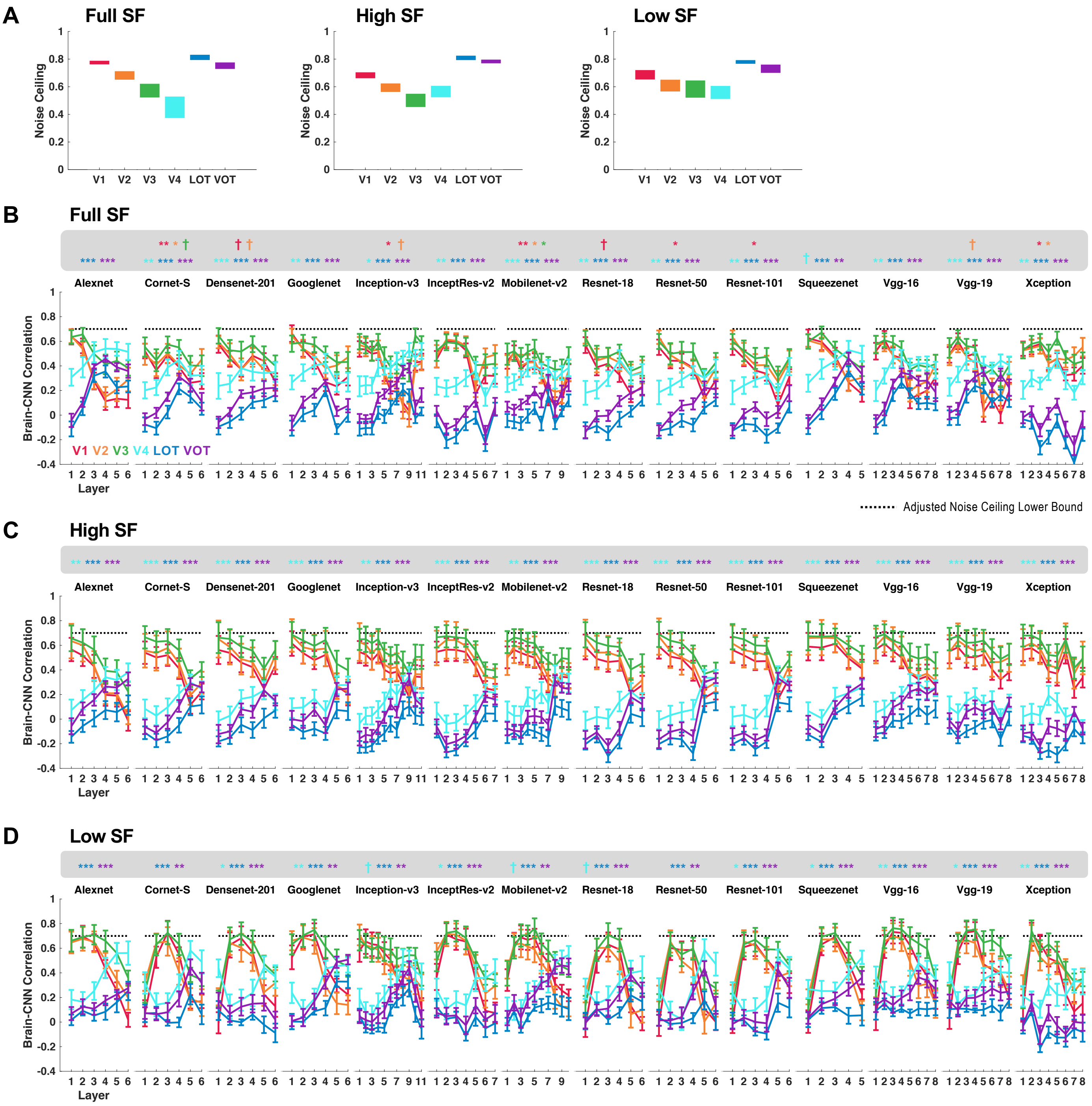

Supplementary Figure 5. Quantifying the brain-CNN correspondence in Experiment 2 with Full, High SF and Low SF images from real-world object categories. **(A)** The upper and lower bounds of noise ceiling of the fMRI responses for each image condition. **(B)** to **(D)** RDM correlations of each brain region with each sampled layer in each CNN for the Full, High SF and Low SF images, respectively. $N = 10$ human participants. For illustration purposes, the lower bounds of the noise ceiling from all brain regions were shifted to 0.7, and the difference between the actual noise ceiling and 0.7 was subtracted from each brain-CNN correlation value. The asterisks at the top of each plot mark the significance levels of the differences between the highest correlations (Fisher-transformed) of brain regions and CNN layers and the corresponding lower bound of the noise ceiling; one-tailed *t*-tests were used, and all *p* values reported were corrected for multiple comparisons for the 6 brain regions included using the Benjamini–Hochberg procedure. Error bars indicate standard errors of the means. † $p < .1$, * $p < .05$, ** $p < .01$, *** $p < .001$. Source data are provided as a Source Data file.

Supplementary Figure 6

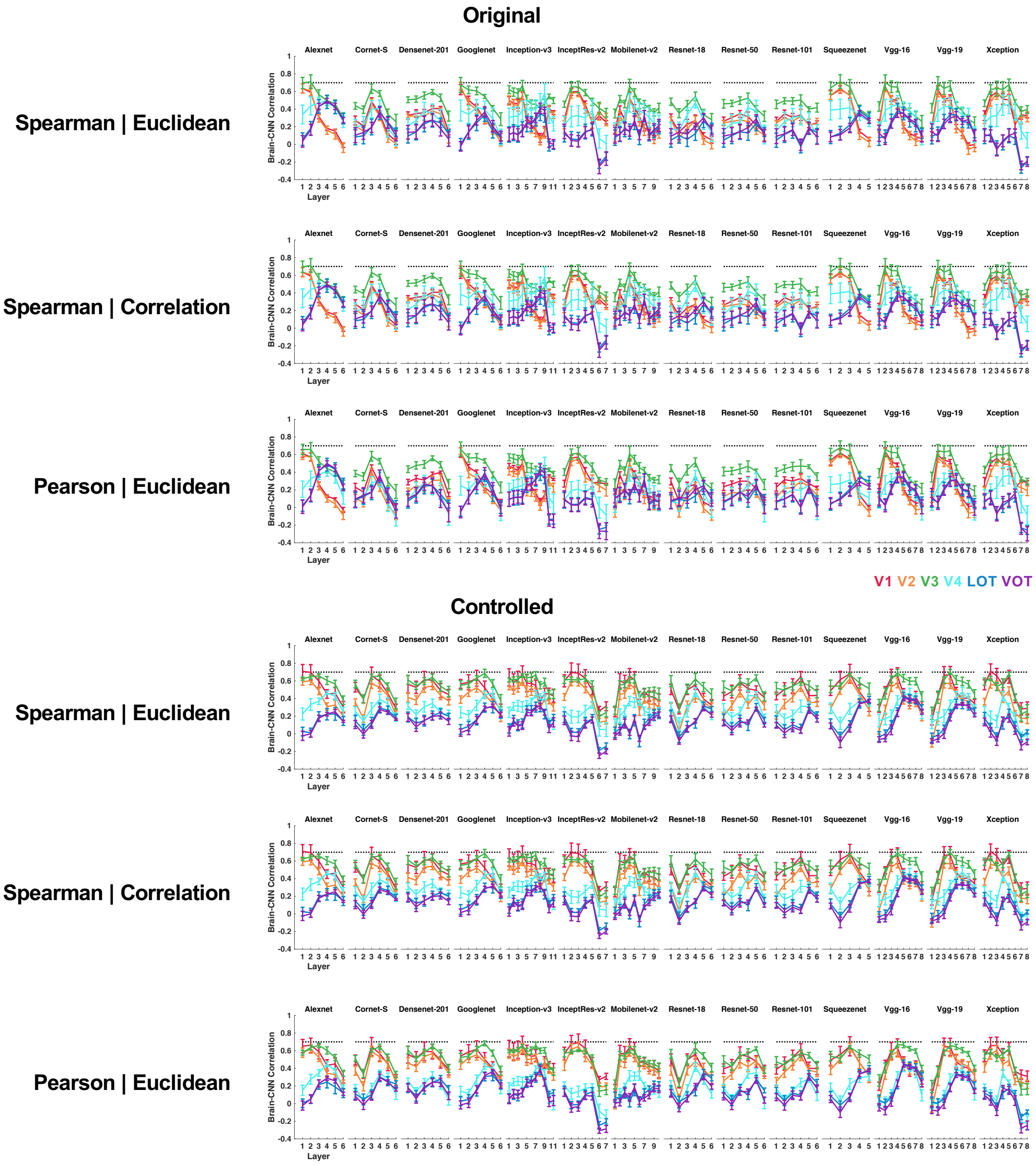

<u>Supplementary Figure 6.</u> Quantifying the brain-CNN correspondence in Experiment 1. Three different measures were used, including using Spearman brain-CNN correlation with Euclidean distance measures for RDM construction (same as those shown in Supplementary Figure 4), using Spearman brain-CNN correlation with correlation measures for RDM construction, and using Pearson brain-CNN correlation with Euclidean distance measures for RDM construction. *N* = 6 human participants. Virtually the same results were obtained in all three measures. For illustration purposes, the lower bounds of the noise ceiling from all brain regions were shifted to 0.7, and the difference between the actual noise ceiling and 0.7 was subtracted from each brain-CNN correlation value. Error bars indicate standard errors of the means. Source data are provided as a Source Data file.

Supplementary Figure 7

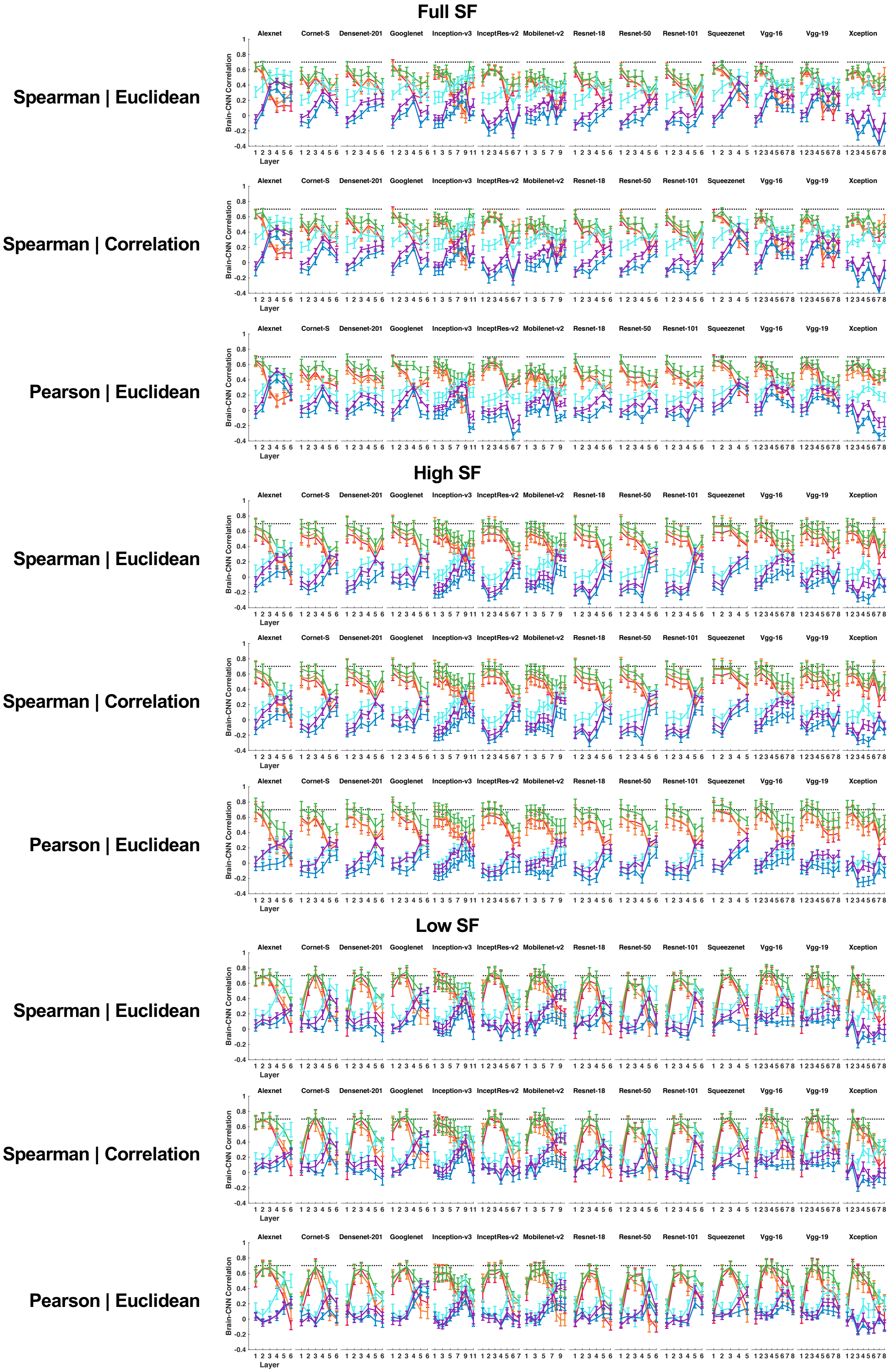

Supplementary Figure 7. Quantifying the brain-CNN correspondence in Experiment 2. Three different measures were used, including using Spearman brain-CNN correlation with Euclidean distance measures for RDM construction (same as those shown in Supplementary Figure 5), using Spearman brain-CNN correlation with correlation measures for RDM construction, and using Pearson brain-CNN correlation with Euclidean distance measures for RDM construction. $N = 10$ human participants. Virtually the same results were obtained in all three measures. For illustration purposes, the lower bounds of the noise ceiling from all brain regions were shifted to 0.7 and the difference between the actual noise ceiling, and 0.7 was subtracted from each brain-CNN correlation value. Error bars indicate standard errors of the means. Source data are provided as a Source Data file.

Supplementary Figure 8

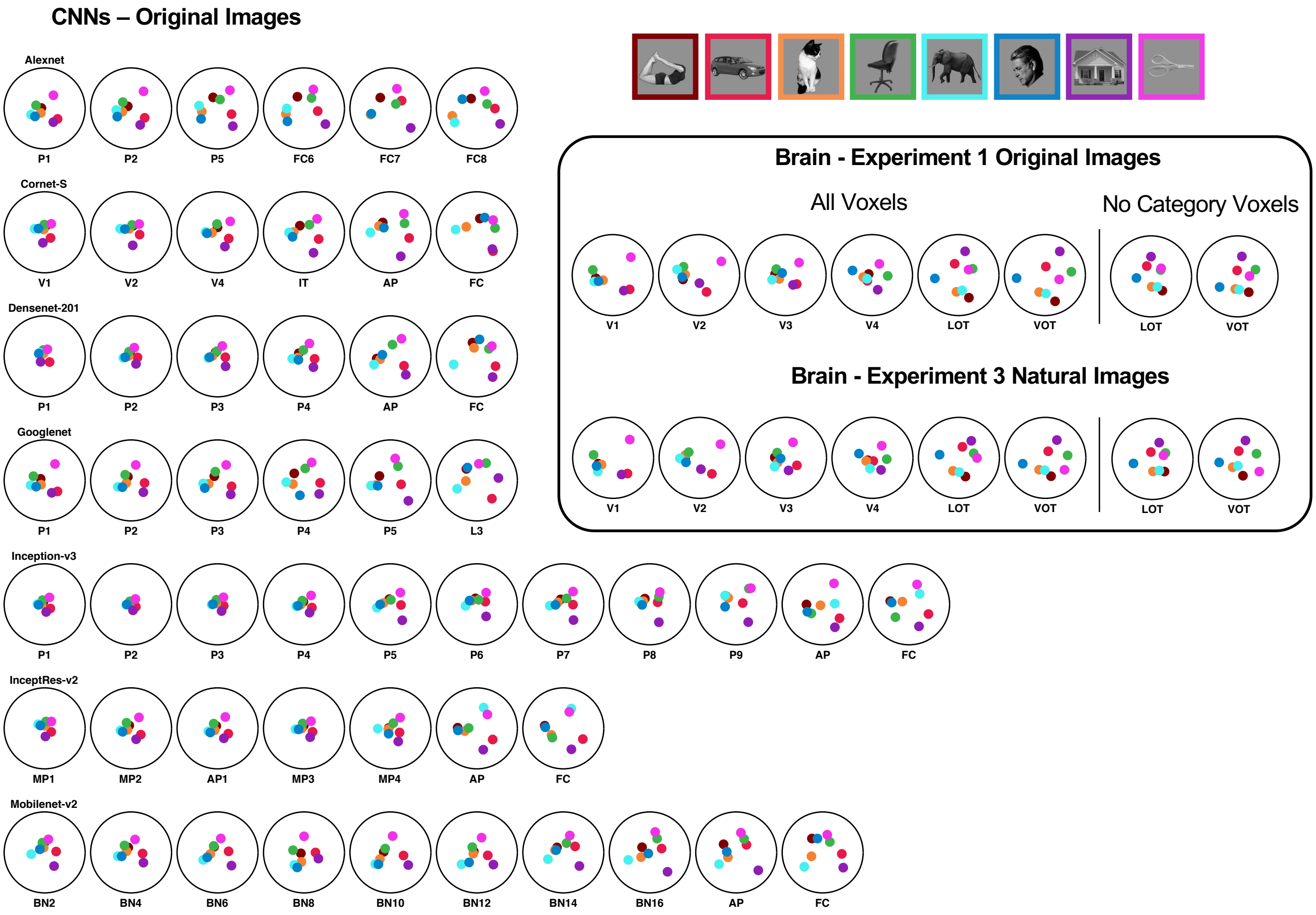

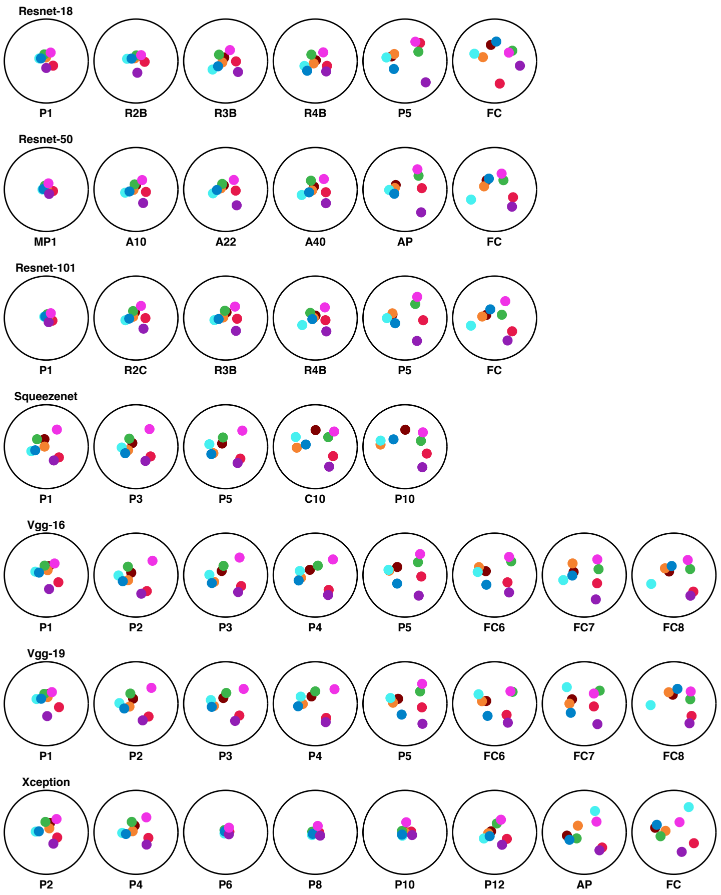

<u>Supplementary Figure 8.</u> Visualizing the representational structures for the original real-world object images in all human visual regions and CNN layers examined. Results for the human visual regions are shown with category-selective voxels either included (All Voxels) or excluded (No Category Voxels). Since rotations and flips preserve distances on these MDS plots, to make these plots more informative and to see how the representational structure evolves across brain regions and CNN layers, we manually rotated and/or flipped each MDS when necessary. Source data are provided as a Source Data file.

Supplementary Figure 9

CNNs – Controlled Images

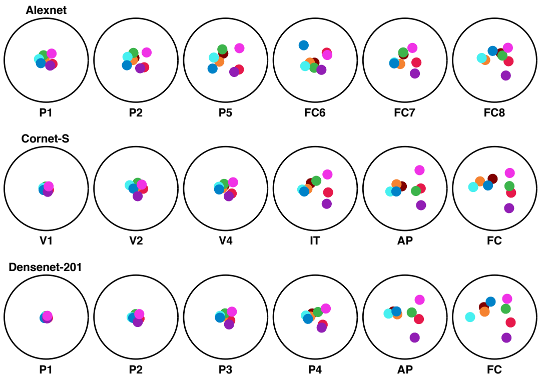

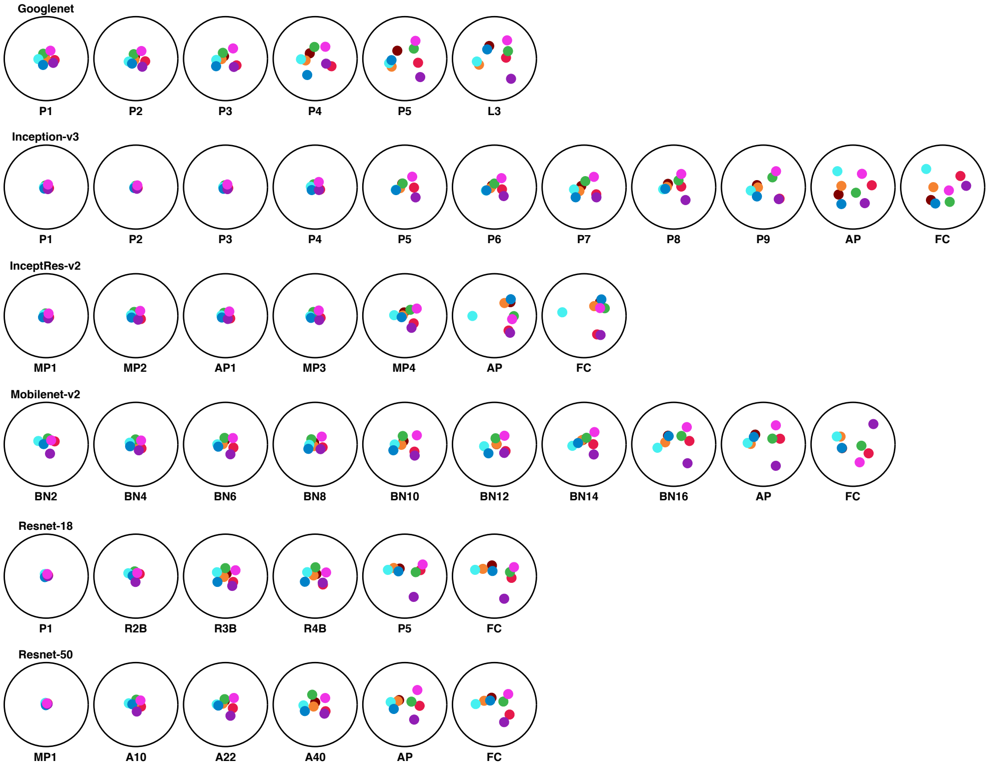

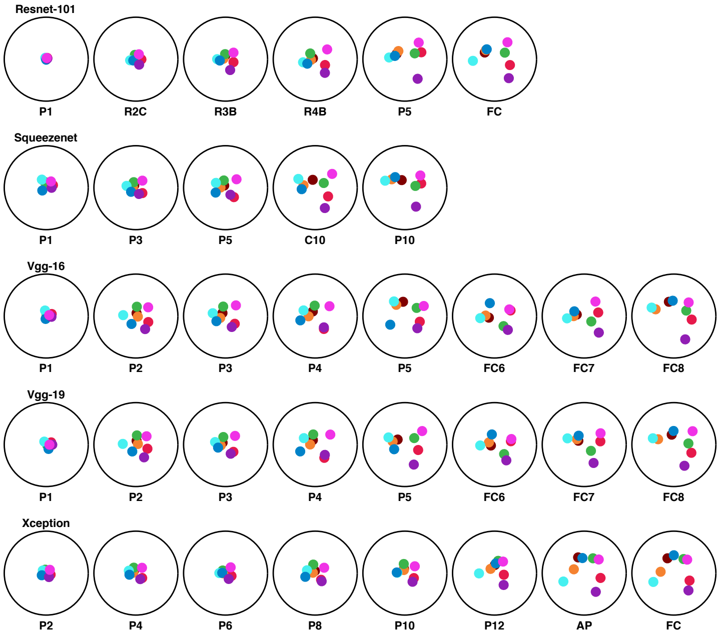

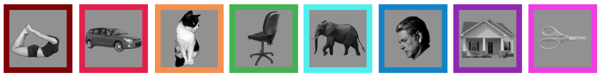

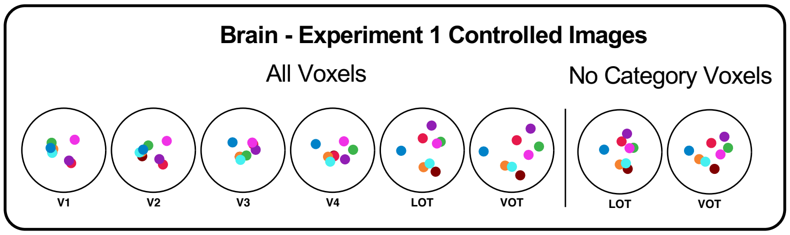

Supplementary Figure 9. Visualizing the representational structures for the controlled real-world object images in all human visual regions and CNN layers examined. Results for the human visual regions are shown with category-selective voxels either included (All Voxels) or excluded (No Category Voxels). Since rotations and flips preserve distances on these MDS plots, to make these plots more informative and to see how the representational structure evolves across brain regions and CNN layers, we manually rotated and/or flipped each MDS when necessary. Source data are provided as a Source Data file.

Supplementary Figure 10

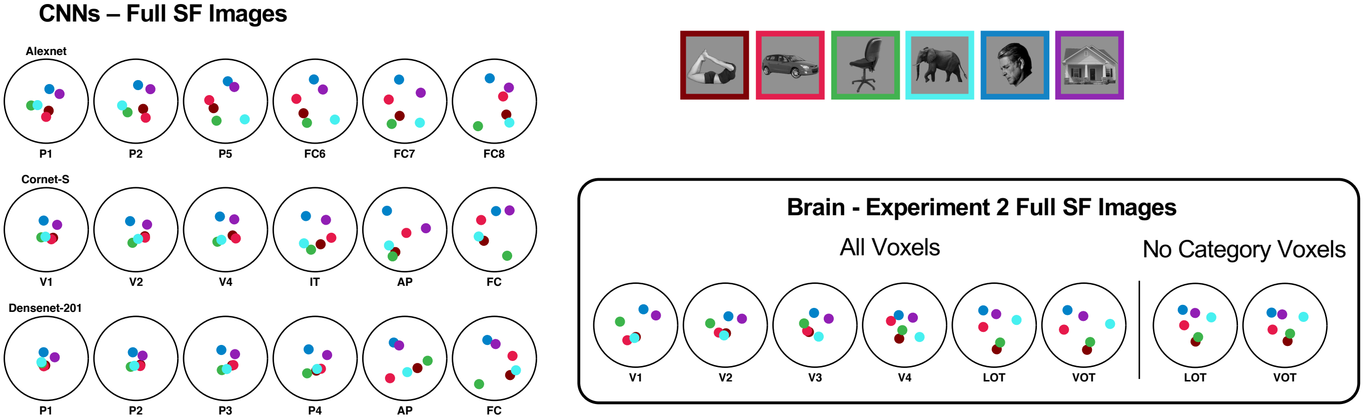

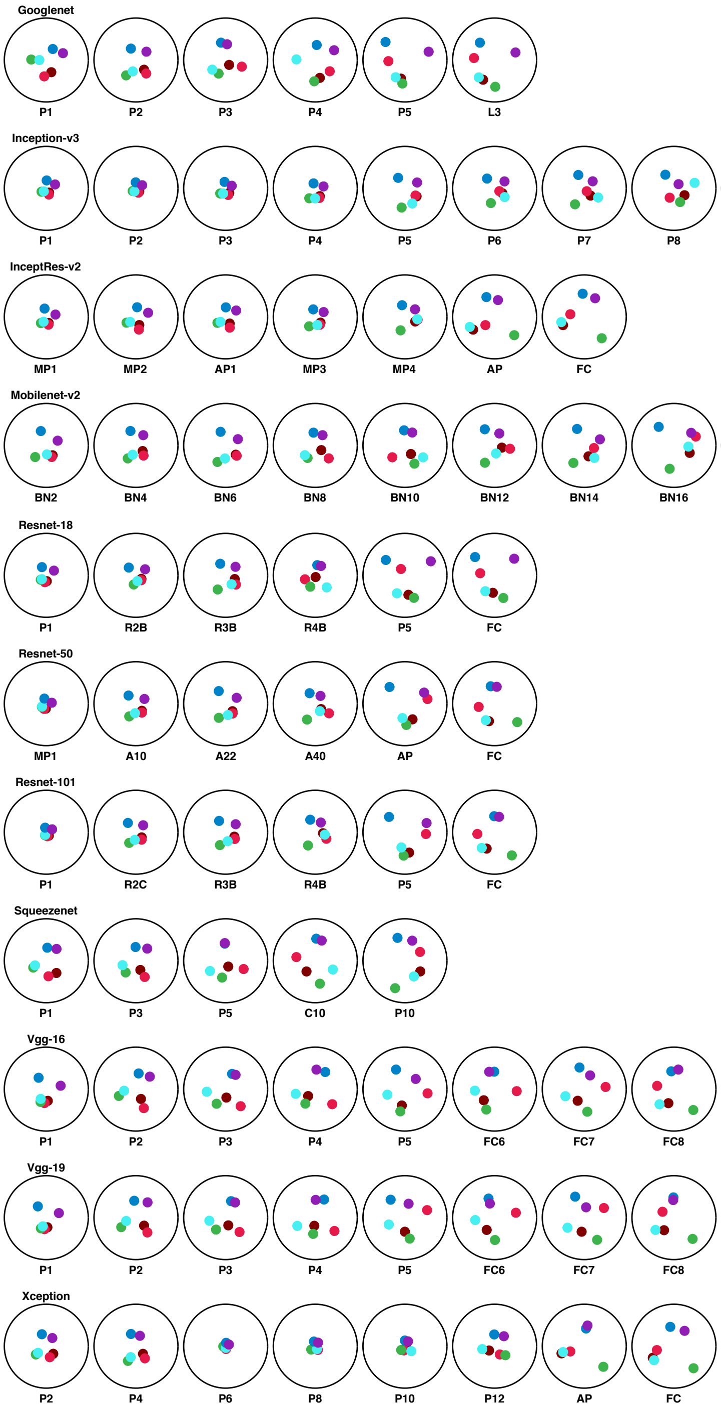

Supplementary Figure 10. Visualizing the representational structures for the full SF real-world object images in all human visual regions and CNN layers examined. Results for the human visual regions are shown with category-selective voxels either included (All Voxels) or excluded (No Category Voxels). Since rotations and flips preserve distances on these MDS plots, to make these plots more informative and to see how the representational structure evolves across brain regions and CNN layers, we manually rotated and/or flipped each MDS when necessary. Source data are provided as a Source Data file.

Supplementary Figure 11

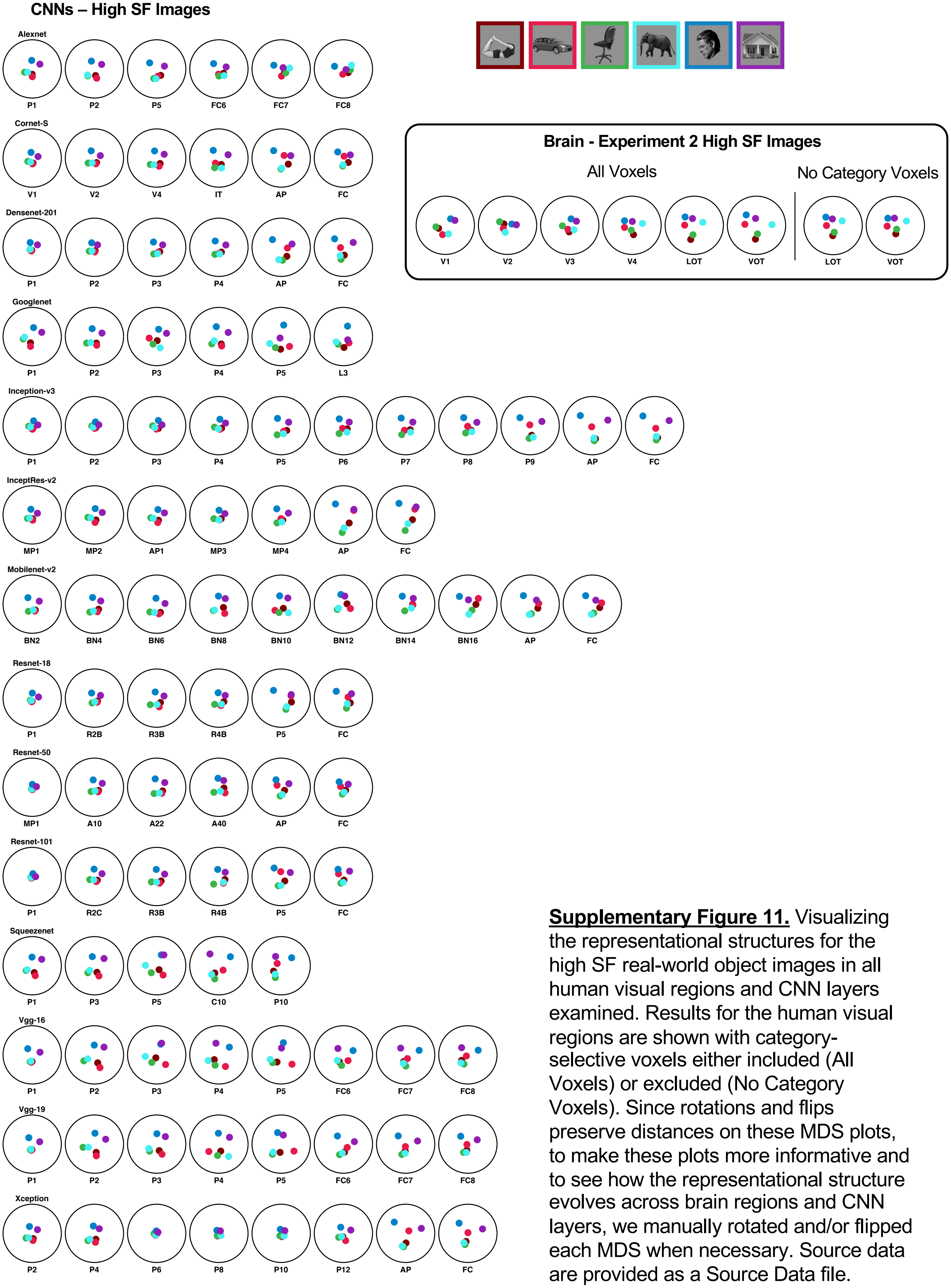

<u>Supplementary Figure 11.</u> Visualizing the representational structures for the high SF real-world object images in all human visual regions and CNN layers examined. Results for the human visual regions are shown with category-selective voxels either included (All Voxels) or excluded (No Category Voxels). Since rotations and flips preserve distances on these MDS plots, to make these plots more informative and to see how the representational structure evolves across brain regions and CNN layers, we manually rotated and/or flipped each MDS when necessary. Source data are provided as a Source Data file.

Supplementary Figure 12

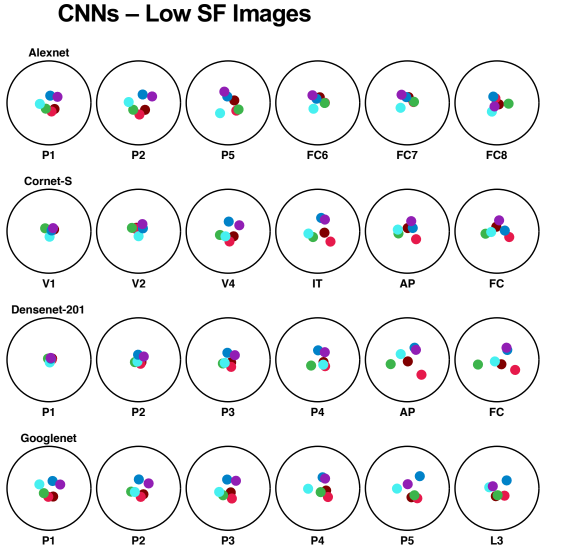

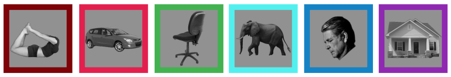

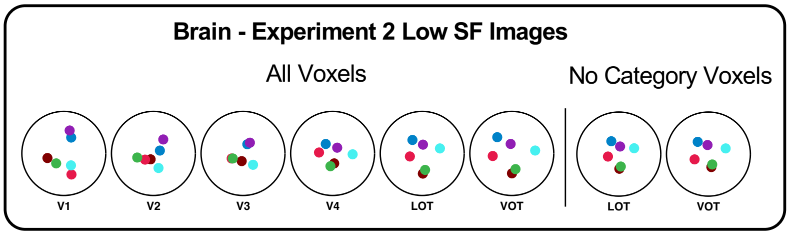

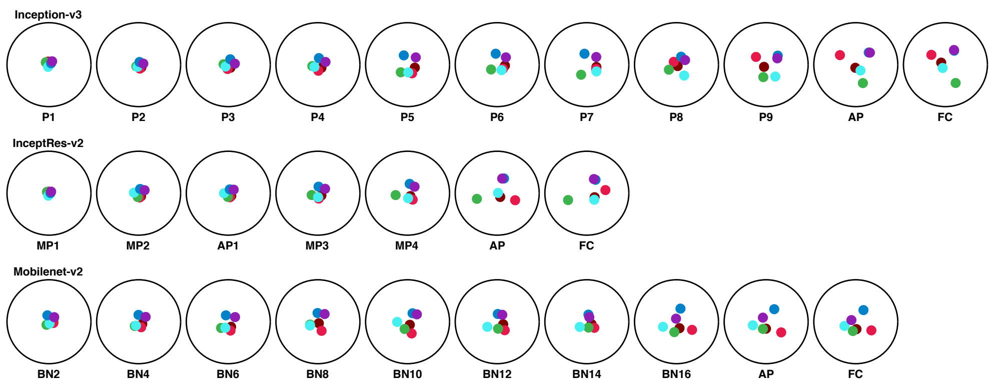

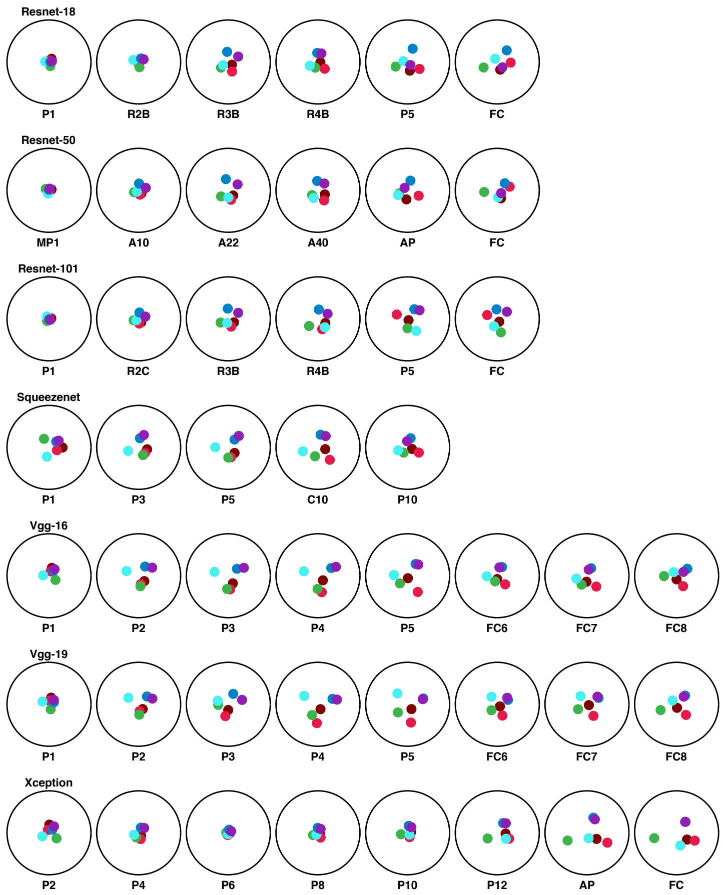

<u>Supplementary Figure 12.</u> Visualizing the representational structures for the low SF real-world object images in all human visual regions and CNN layers examined. Results for the human visual regions are shown with category-selective voxels either included (All Voxels) or excluded (No Category Voxels). Since rotations and flips preserve distances on these MDS plots, to make these plots more informative and to see how the representational structure evolves across brain regions and CNN layers, we manually rotated and/or flipped each MDS when necessary. Source data are provided as a Source Data file.

Supplementary Figure 13

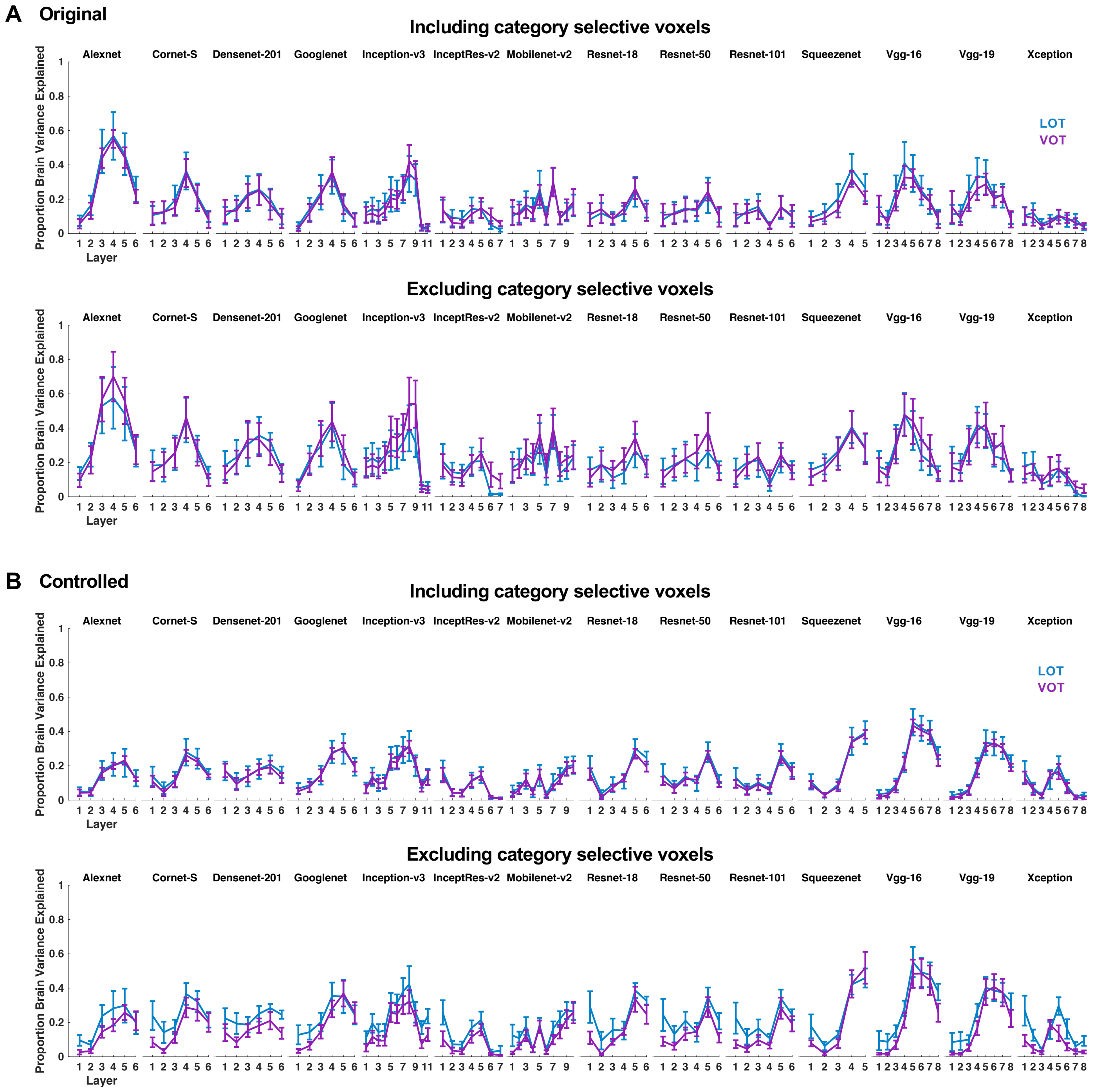

<u>Supplementary Figure 13.</u> The amount of LOT and VOT RDM variance explained by CNNs including and excluding category-selective voxels in LOT and VOT for Experiment 1 with real-world object images. $N = 6$ human participants. Within each CNN, the maximum amount of variance explained for each brain region (i.e., the peak value) was compared between when category-selective voxels were included and when they were excluded. **(A)** Results from the original images. No differences were found. **(B)** Results from the controlled images. In LOT, differences were found for InceptRes-v2, $p = .005$; and Xception, $p < .001$. No other differences were found. Two-tailed t-tests were used, and all p values reported were uncorrected. Error bars indicate standard errors of the means. Source data are provided as a Source Data file.

Supplementary Figure 14

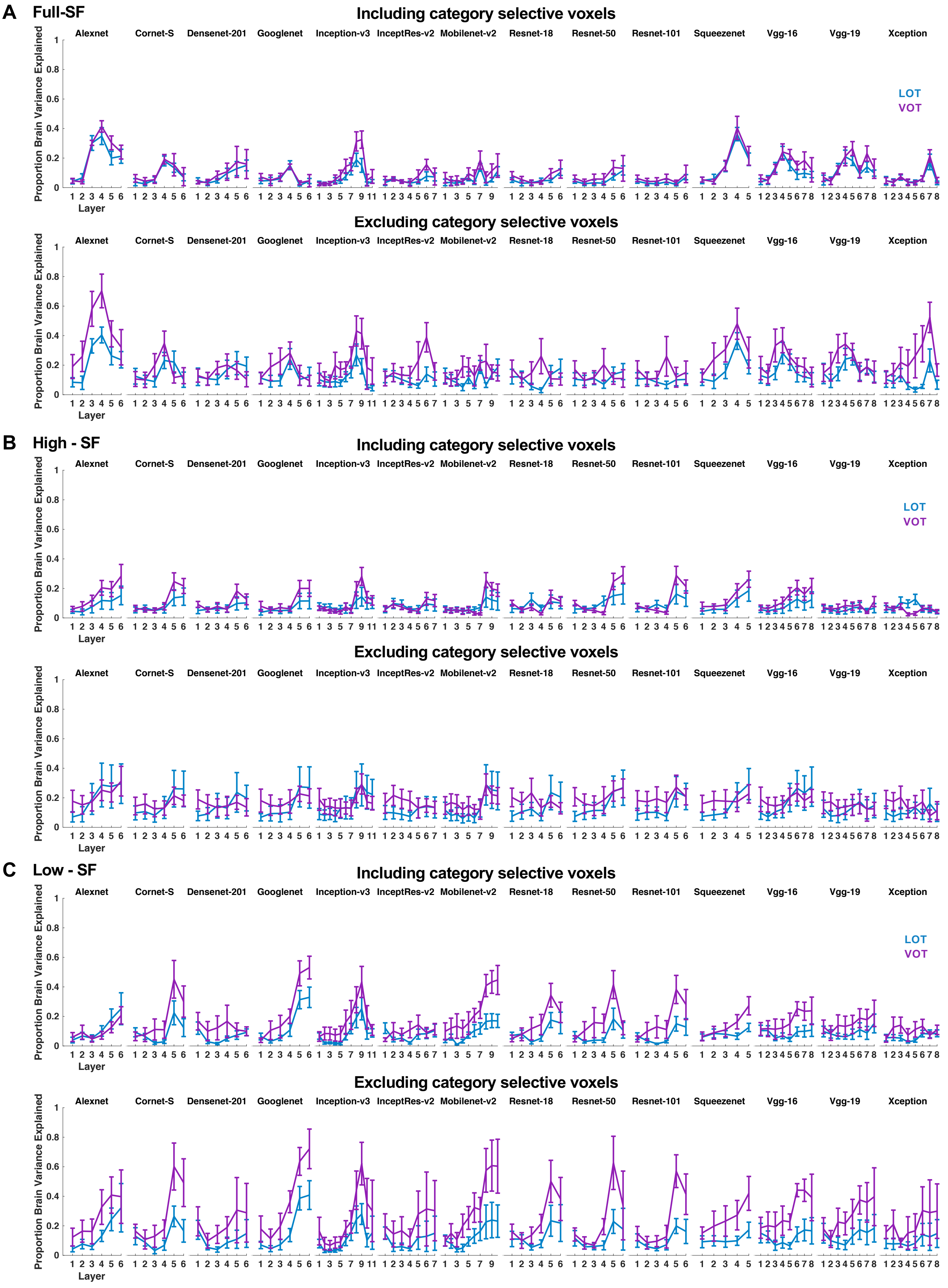

<u>Supplementary Figure 14.</u> The amount of LOT and VOT RDM variance explained by CNNs including and excluding category-selective voxels in LOT and VOT for Experiment 2 with real-world object images. $N = 10$ human participants. Within each CNN, the maximum amount of variance explained for each brain region (i.e., the peak value) was compared between when category-selective voxels were included and when they were excluded. **(A)** Results from the full-SF images. In LOT difference was found for InceptRes-v2, $p = .02$. In VOT differences were found for Alexnet, $p = .02$; InceptRes-v2, $p = .03$; and Xception, $p = .02$. **(B)** Results from high-SF images. In LOT, differences were found for Squeezenet, $p = .03$. **(C)** Results from low-SF image. In VOT, differences were found for Resnet-101, $p = .03$; and Vgg-16, $p = .001$. No other differences were found. Two-tailed *t*-tests were used, and all *p* values reported were uncorrected. Error bars indicate standard errors of the means. Source data are provided as a Source Data file.

Supplementary Figure 15

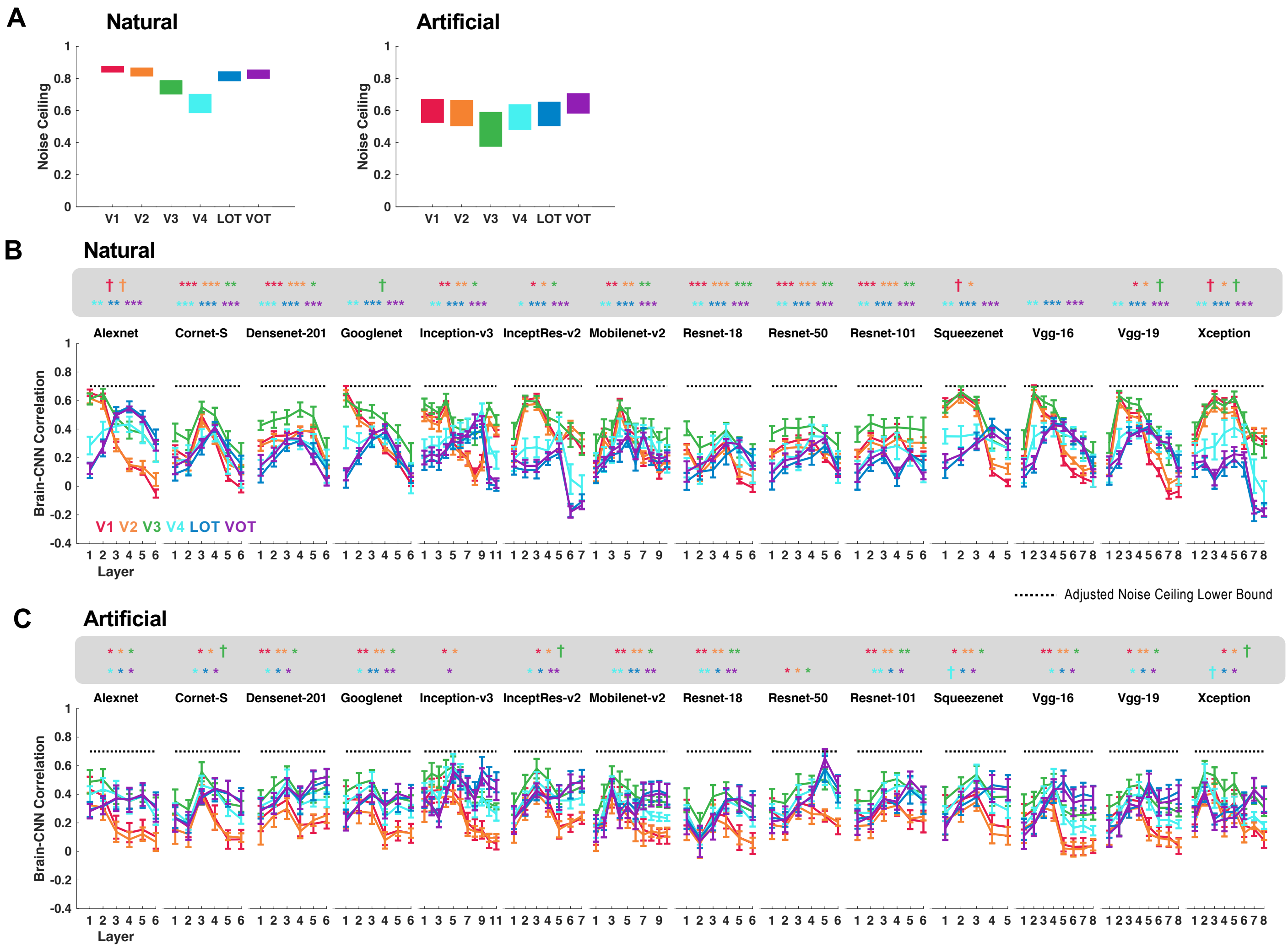

Supplementary Figure 15. Quantifying the brain-CNN correspondence in Experiment 3 with images from real-world and artificial object categories. **(A)** The upper and lower bounds of noise ceiling of the fMRI responses for each image condition. **(B)** and **(C)** RDM correlations of each brain region with each sampled layer in each CNN for the real-world and artificial object images, respectively. N = 6 human participants. For illustration purposes, the lower bounds of the noise ceiling from all brain regions were shifted to 0.7, and the difference between the actual noise ceiling and 0.7 was subtracted from each brain-CNN correlation value. The asterisks at the top of each plot mark the significance levels of the differences between the highest correlations (Fisher-transformed) of brain regions and CNN layers and the corresponding lower bound of the noise ceiling; one-tailed t-tests were used, and all p values reported were corrected for multiple comparisons for the 6 brain regions included using the Benjamini–Hochberg procedure. Error bars indicate standard errors of the means. † $p < .1$, * $p < .05$, ** $p < .01$, *** $p < .001$. Source data are provided as a Source Data file. (We note that the lower bounds of the noise ceiling were higher for natural than artificial object categories. This could be due to several reasons. First, visual neurons may be more responsive to natural than artificial objects, as our brain has evolved to process natural rather than artificial stimuli. Second, the natural images were overall more variable in identity within a category and thus more interesting to look at than the similar-looking artificial images. This could potentially increase SNR for the natural objects. Lastly, the natural categories were more distinctive from each other than the artificial ones were. Thus any distortion in the representational structure due to noise would be smaller for the natural than for the artificial objects, potentially increasing RDM consistency among the subjects.)

Supplementary Figure 16

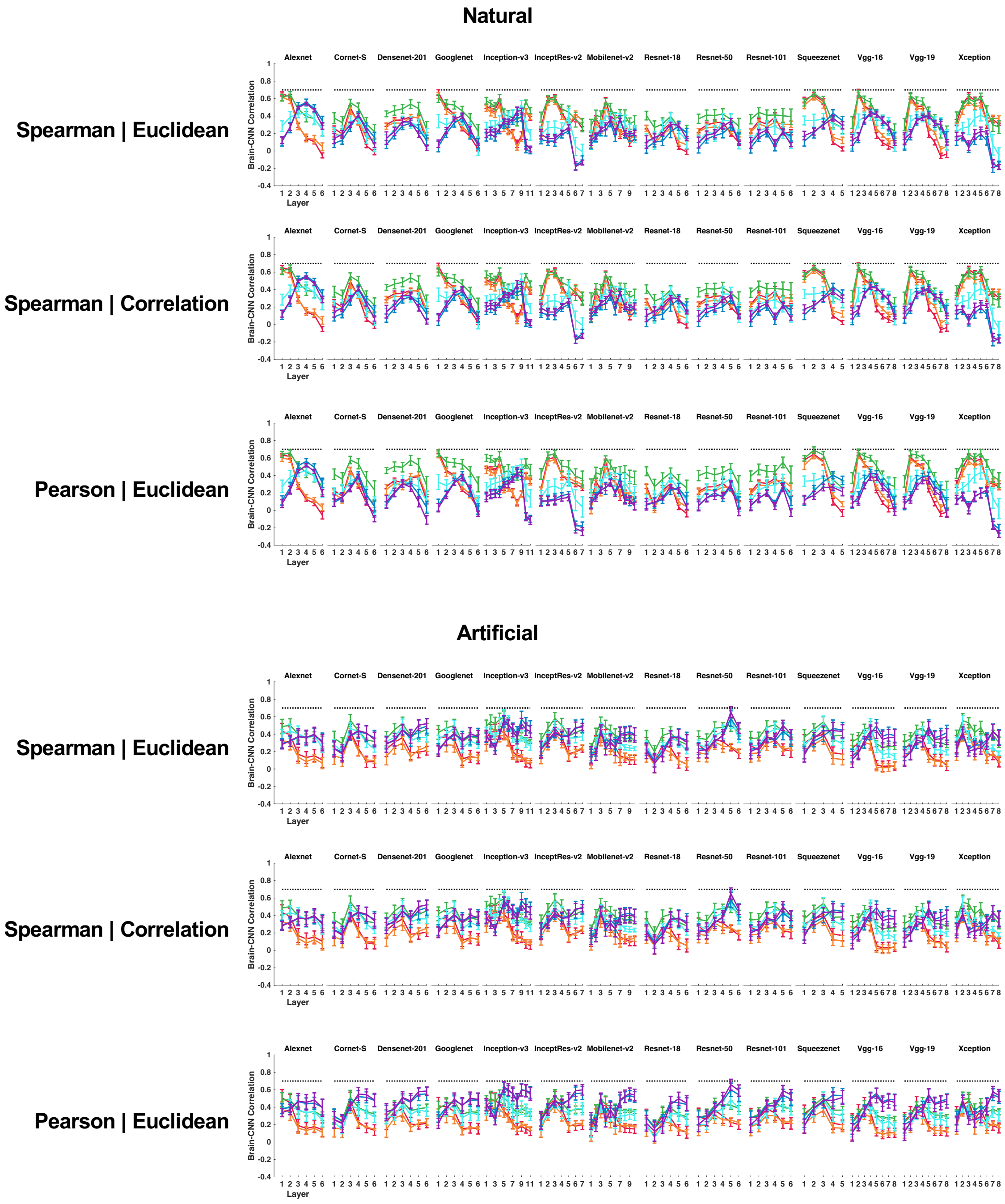

Supplementary Figure 16. Quantifying the brain-CNN correspondence in Experiment 3. Three different measures were used, including using Spearman brain-CNN correlation with Euclidean distance measures for RDM construction (same as those shown in Supplementary Figure 15), using Spearman brain-CNN correlation with correlation measures for RDM construction, and using Pearson brain-CNN correlation with Euclidean distance measures for RDM construction. *N* = 6 human participants. Virtually the same results were obtained in all three measures. For illustration purposes, the lower bounds of the noise ceiling from all brain regions were shifted to 0.7, and the difference between the actual noise ceiling and 0.7 was subtracted from each brain-CNN correlation value. Error bars indicate standard errors of the means. Source data are provided as a Source Data file.

Supplementary Figure 17

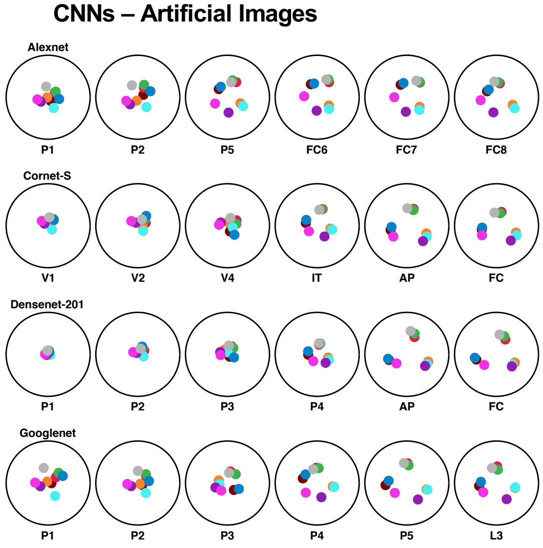

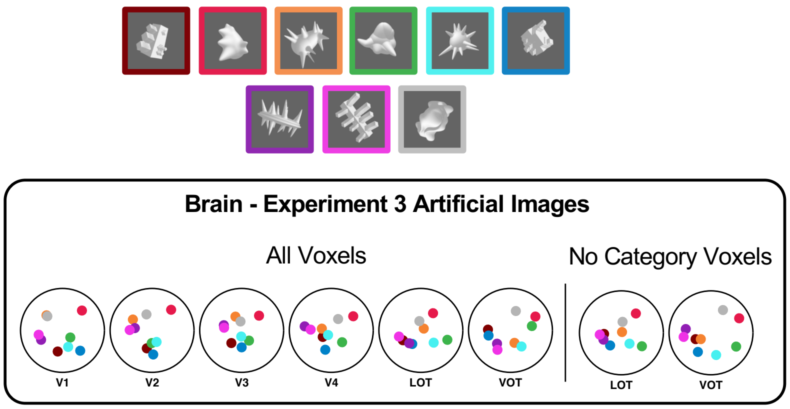

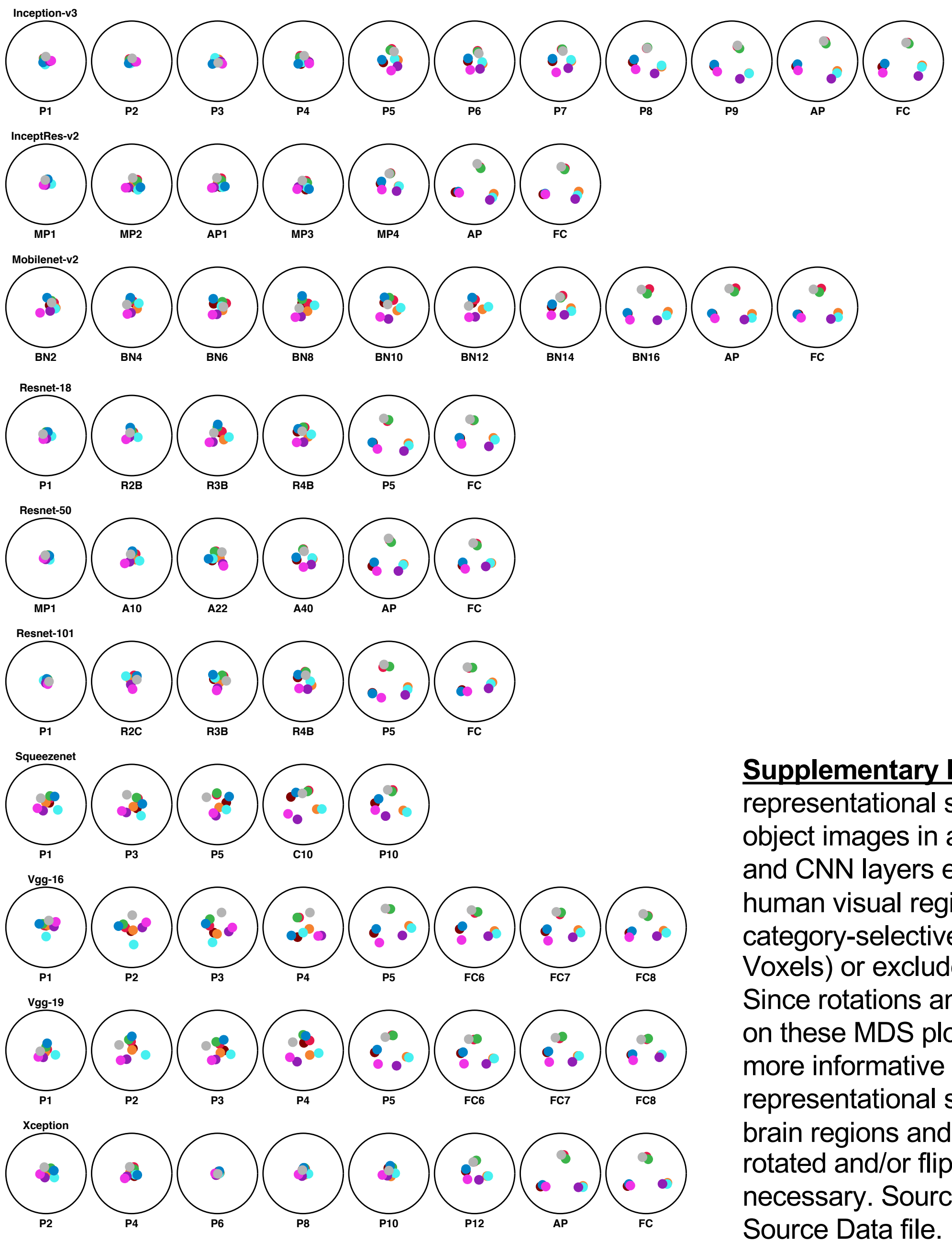

Supplementary Figure 17. Visualizing the representational structures for the artificial object images in all human visual regions and CNN layers examined. Results for the human visual regions are shown with category-selective voxels either included (All Voxels) or excluded (No Category Voxels). Since rotations and flips preserve distances on these MDS plots, to make these plots more informative and to see how the representational structure evolved across brain regions and CNN layers, we manually rotated and/or flipped each MDS when necessary. Source data are provided as a Source Data file.

Supplementary Figure 18

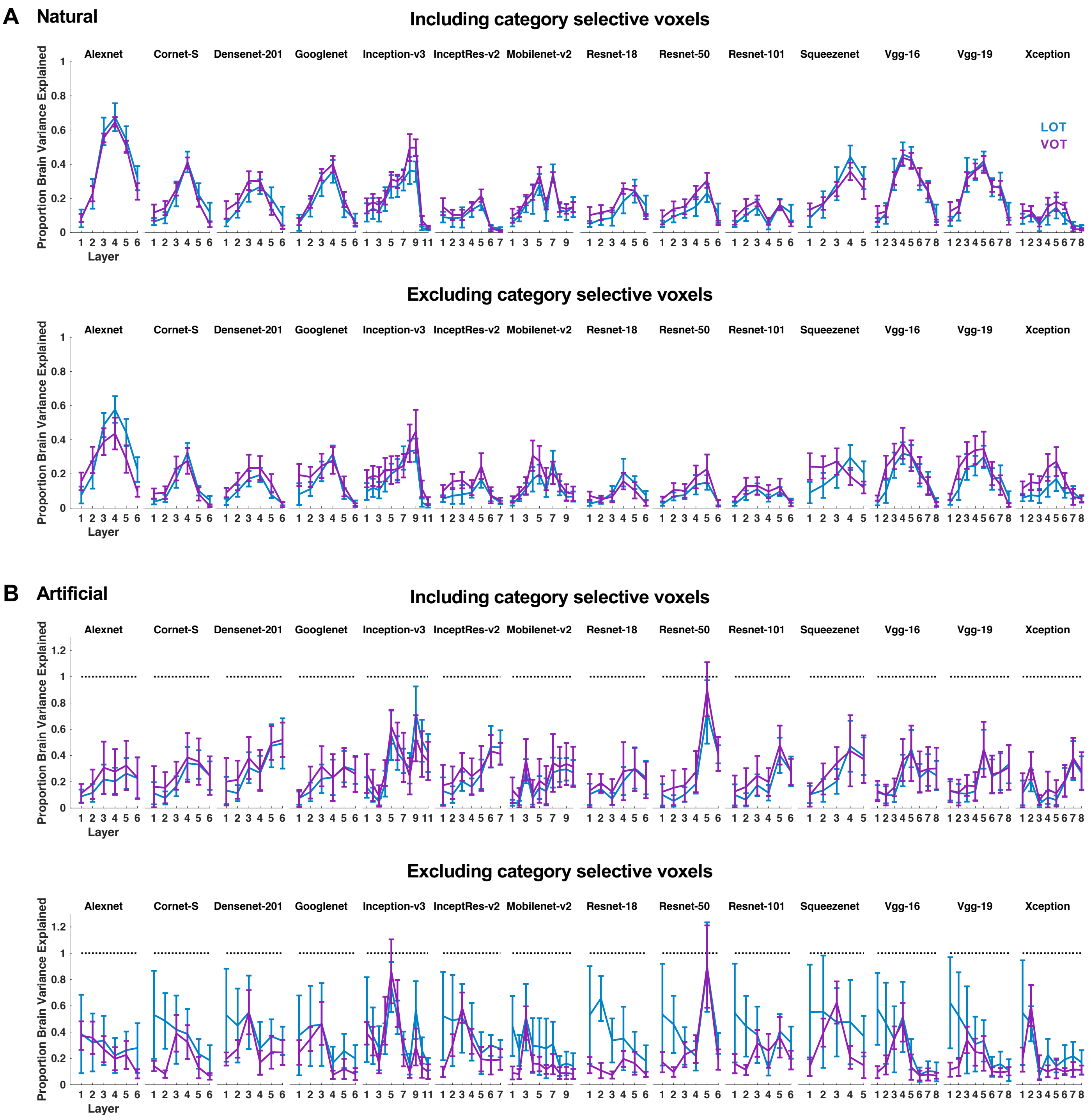

Supplementary Figure 18. The amount of LOT and VOT RDM variance explained by CNNs including and excluding category-selective voxels in LOT and VOT for Experiment 3. $N = 6$ human participants. Within each CNN, the maximum amount of variance explained for each brain region (i.e., the peak value) was compared between when category-selective voxels were included and when they were excluded. **(A)** Results from the real-world natural object images. In VOT, differences were found for Resnet-18, $p = .04$. No other comparisons reached significance. **(B)** Results from the artificial object images. No difference reached significance. Two-tailed t-tests were used, and all p values reported were uncorrected. Error bars indicate standard errors of the means. Source data are provided as a Source Data file.

Supplementary Figure 19

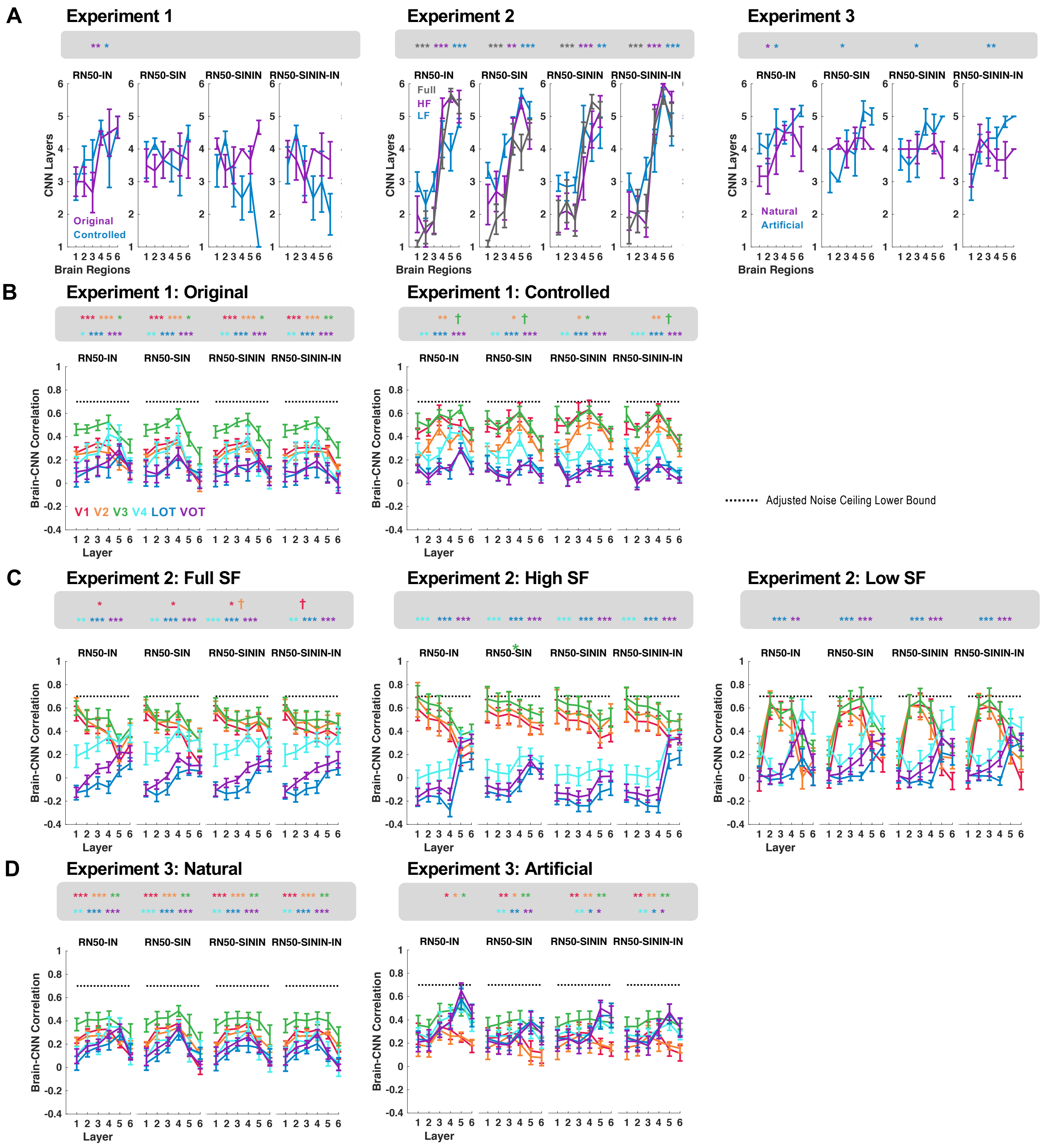

<u>Supplementary Figure 19.</u> Comparing Resnet-50 trained with original and stylized ImageNet images with brain responses from Experiments 1 to 3. **(A)** The correspondence between the brain and CNN in their representational structure. **(B)** to **(D)** RDM correlations between brain regions and CNN layers. *N* = 6, 10 and 6 human participants, respectively, for Experiments 1 to 3. Resnet-50 was pretrained either with the original ImageNet images (RN50-IN), the stylized ImageNet Images (RN50-SIN), both the original and the stylized ImageNet Images (RN50-SININ), or both sets of images and then fine-tuned with the stylized ImageNet images (RN50-SININ-IN). **(A)** The averaged CNN layer numbers across the human participants that showed the greatest RDM correlation for each brain region in each experimental condition, with the error bars indicating the standard errors of the mean across participants. The asterisks at the top of each plot mark the significance levels of the brain-CNN correspondence. One-tailed *t*-tests were used, and all *p* values reported were corrected for multiple comparisons for the number of image conditions included in an experiment using the Benjamini–Hochberg procedure. In **(B)** to **(D)**, for illustration purposes, the lower bounds of the noise ceiling from all brain regions were shifted to 0.7, and the difference between the actual noise ceiling and 0.7 was subtracted from each brain-CNN correlation value. The asterisks at the top of each plot mark the significance levels of the differences between the highest correlations (Fisher-transformed) of brain regions and CNN layers and the corresponding lower bound of the noise ceiling; one-tailed *t*-tests were used, and all *p* values reported were corrected for multiple comparisons for the 6 brain regions included using the Benjamini–Hochberg procedure. Error bars indicate standard errors of the means. † $p < .1$, * $p < .05$, ** $p < .01$, *** $p < .001$. Source data are provided as a Source Data file.
