# Supplementary material for: Limits to visual representational correspondence between convolutional neural networks and the human brain
Source: Nat Commun. 2021 Apr 6;12:2065. doi: 10.1038/s41467-021-22244-7 (PMC8024324; doi:10.1038/s41467-021-22244-7)
Supplement: Supplementary file 3 — Reporting Summary [file 41467_2021_22244_MOESM3_ESM.pdf]

nature research

Corresponding author(s): Yaoda Xu

Last updated by author(s): Feb 8, 2021

Reporting Summary

Nature Research wishes to improve the reproducibility of the work that we publish. This form provides structure for consistency and transparency in reporting. For further information on Nature Research policies, see Authors & Referees and the Editorial Policy Checklist .

Statistics

For all statistical analyses, confirm that the following items are present in the figure legend, table legend, main text, or Methods section.

n/a	Confirmed	
☐	☒	The exact sample size (*n*) for each experimental group/condition, given as a discrete number and unit of measurement
☐	☒	A statement on whether measurements were taken from distinct samples or whether the same sample was measured repeatedly
☐	☒	The statistical test(s) used AND whether they are one- or two-sided<br>*Only common tests should be described solely by name; describe more complex techniques in the Methods section.*
☐	☒	A description of all covariates tested
☐	☒	A description of any assumptions or corrections, such as tests of normality and adjustment for multiple comparisons
☐	☒	A full description of the statistical parameters including central tendency (e.g. means) or other basic estimates (e.g. regression coefficient) AND variation (e.g. standard deviation) or associated estimates of uncertainty (e.g. confidence intervals)
☐	☒	For null hypothesis testing, the test statistic (e.g. *F*, *t*, *r*) with confidence intervals, effect sizes, degrees of freedom and *P* value noted<br>*Give P values as exact values whenever suitable.*
☒	☐	For Bayesian analysis, information on the choice of priors and Markov chain Monte Carlo settings
☒	☐	For hierarchical and complex designs, identification of the appropriate level for tests and full reporting of outcomes
☐	☒	Estimates of effect sizes (e.g. Cohen's *d*, Pearson's *r*), indicating how they were calculated

Our web collection on statistics for biologists contains articles on many of the points above.

Software and code

Policy information about availability of computer code

Data collection	Matlab (version R2019a), Python (version 3.7)
Data analysis	FreeSurfer (version 6, which includes Fs-Fast), Matlab (version R2019a), Python (version 3.7), Matlab SHINE Toolbox (Willenbockel et al., 2010)

For manuscripts utilizing custom algorithms or software that are central to the research but not yet described in published literature, software must be made available to editors/reviewers. We strongly encourage code deposition in a community repository (e.g. GitHub). See the Nature Research guidelines for submitting code & software for further information.

Data

Policy information about availability of data

All manuscripts must include a data availability statement. This statement should provide the following information, where applicable:

- Accession codes, unique identifiers, or web links for publicly available datasets
- A list of figures that have associated raw data
- A description of any restrictions on data availability

Data supporting the findings of this study are publicly available at https://osf.io/tsz47/.

Field-specific reporting

Please select the one below that is the best fit for your research. If you are not sure, read the appropriate sections before making your selection.

☒ Life sciences ☐ Behavioural & social sciences ☐ Ecological, evolutionary & environmental sciences

Life sciences study design

All studies must disclose on these points even when the disclosure is negative.

Sample size	Sample sizes were chosen based on the typical sample sizes used in previously published studies that are similar to the present study (e.g., Haxby et al., 2001; Kamitani & Tong, 2005).
Data exclusions	None.
Replication	The main results were successfully replicated across three independent sets of data.
Randomization	We used a within-subject design and as such randomization across subjects was not needed.
Blinding	Blinding was not necessary as the experimenters' knowledge of participant assignment during data collection and analysis do not influence the outcome of the results.

Reporting for specific materials, systems and methods

We require information from authors about some types of materials, experimental systems and methods used in many studies. Here, indicate whether each material, system or method listed is relevant to your study. If you are not sure if a list item applies to your research, read the appropriate section before selecting a response.

Materials & experimental systems

n/a	Involved in the study	
☒	☐	Antibodies
☒	☐	Eukaryotic cell lines
☒	☐	Palaeontology
☒	☐	Animals and other organisms
☐	☒	Human research participants
☒	☐	Clinical data

Methods

n/a	Involved in the study	
☒	☐	ChIP-seq
☒	☐	Flow cytometry
☐	☒	MRI-based neuroimaging

Human research participants

Policy information about studies involving human research participants

Population characteristics	Healthy human participants (about 50% female) with normal or corrected to normal visual acuity, all right-handed, and aged between 18 and 35 took part in the experiments.
Recruitment	Participants were recruited from the Harvard University community. There is no potential self-selection bias or other biases that could have impacted the results.
Ethics oversight	The experiments were approved by the Committee on the Use of Human Subjects at Harvard University.

Note that full information on the approval of the study protocol must also be provided in the manuscript.

Magnetic resonance imaging

Experimental design

Design type	task state; block design
Design specifications	In Experiment 1, we used cut-out grey-scaled images from eight real-world object categories (faces, bodies, houses, cats, elephants, cars, chairs, and scissors) and modified them to occupy roughly the same area on the screen. For each object category, we selected ten exemplar images that varied in identity, pose and viewing angle to minimize the low-level similarities among them. In the original image condition, unaltered images were shown. In the controlled image condition, images were shown with contrast, luminance and spatial frequency equalized across all the categories using the SHINE toolbox (Willenbockel et al., 2010). Participants fixated at a central red dot throughout the experiment. Eye-movements were monitored in all the fMRI experiments to ensure proper fixation. During the experiment, blocks of images were shown. Each block contained a random sequential presentation of ten exemplars from the same object category. Each image was presented for 200 msec followed by a 600 msec blank interval between the images. Participants detected a one-back repetition of the exact same image. This task engaged participants' attention on the object shapes and ensured robust fMRI responses. Two image repetitions occurred randomly in each image block. Each experimental run contained 16 blocks, one for each of the 8 categories in each image condition (original or controlled). The order of the eight object categories and the two image conditions were counterbalanced across runs and

participants. Each block lasted 8 secs and followed by an 8-sec fixation period. There was an additional 8-sec fixation period at the beginning of the run. Each participant completed one scan session with 16 runs for this experiment, each lasting 4 mins 24 secs. In Experiment 2, only six of the original eight object categories were included and they were faces, bodies, houses, elephants, cars, and chairs. Images were shown in 3 conditions: Full-SF, High-SF, and Low-SF. In the Full-SF condition, the full spectrum images were shown without modification of the SF content. In the High-SF condition, images were high-pass filtered using an FIR filter with a cutoff frequency of 4.40 cycles per degree. In the Low-SF condition, the images were low-pass filtered using an FIR filter with a cutoff frequency of 0.62 cycles per degree. The DC component was restored after filtering so that the image backgrounds were equal in luminance. Each run contained 18 blocks, one for each of the category and SF condition combination. Each participant completed a single scan session containing 18 experimental runs, each lasting 5 minutes. Other details of the experiment design were identical to that Experiment 1. In Experiment 3, we used unaltered images from both real-world and artificial object categories. The real-world categories were the same eight categories used in Experiment 1. The artificial object categories were nine categories of computer-generated 3D shapes (ten images per category) adopted from Op de Beeck et al. (2008) and shown in random orientations to increase image variation within a category. Each run of the experiment contained 17 stimulus blocks, one for each object category (either real-world or artificial). Each participant completed 18 runs, each lasting 4 mins 40 secs. Other details of the experiment design were identical to that Experiment 1.

Behavioral performance measures

Correct button presses were recorded and were manually checked during the MRI scan to make sure participants were awake and alert during the scan.

Acquisition

Imaging type(s)

functional

Field strength

3T

Sequence & imaging parameters

For all the fMRI scans, a T2*-weighted gradient echo pulse sequence was used. For the experimental scans, 33 axial slices parallel to the AC-PC line (3mm thick, 3 × 3mm2 in-plane resolution with 20% skip) were used to cover the whole brain (TR = 2 s, TE = 29 ms, flip angle = 90°, matrix = 64 × 64). For the LOT/ VOT localizer, 30–31 axial slices parallel to the AC-PC line (3mm thick, 3 × 3mm2 in-plane resolution with no skip) were used to cover occipital, temporal and parts of parietal and frontal lobes (TR = 2 s, TE = 30 ms, flip angle = 90°, matrix = 72 × 72). For topographic mapping 42 slices (3mm thick, 3.125 × 3.125mm2 inplane resolution with no skip) just off parallel to the AC-PC line were collected to cover the whole brain (TR = 2.6 s, TE = 30 ms, flip angle = 90°, matrix = 64 × 64). Different slice prescriptions were used here for the different localizers to be consistent with the parameters used in our previous studies. Because the localizer data were projected into the volume view and then onto individual participants' flattened cortical surface, the exact slice prescriptions used had minimal impact on the final results.

Area of acquisition

Whole brain with functional localizer to define specific areas of interests

Diffusion MRI ☐ Used ☒ Not used

Preprocessing

Preprocessing software

Freesurfer

Normalization

No normalization was applied.

Normalization template

No normalization was applied.

Noise and artifact removal

FMRI data preprocessing included 3D motion correction, slice timing correction and linear and quadratic trend removal.

Volume censoring

No volume censoring was applied.

Statistical modeling & inference

Model type and settings

Used an independent functional localizer-based approach to localize regions of interests; extracted responses from the 75 most reliable voxels from each region; examined z-normalized averaged fMRI response patterns for each condition across runs.

Effect(s) tested

Tested correlations of the representational structures between brain regions and convolutional neural networks.

Specify type of analysis: ☐ Whole brain ☒ ROI-based ☐ Both

Anatomical location(s)

Functional localizers were used.

Statistic type for inference
(See Eklund et al. 2016)

Used an independent functional localizer-based approach, so voxel cluster threshold is not needed.

Correction

FDR correction was applied to the statistical tests conducted.

Models & analysis

n/a	Involved in the study
☒	☐ Functional and/or effective connectivity
☒	☐ Graph analysis
☒	☐ Multivariate modeling or predictive analysis
